# Comparison of the RNA-amplification based methods RT–PCR and NASBA for the detection of circulating tumour cells

**DOI:** 10.1038/sj.bjc.6600014

**Published:** 2002-01-07

**Authors:** S A Burchill, L Perebolte, C Johnston, B Top, P Selby

**Affiliations:** Children's Cancer Research Laboratory, St. James's University Hospital, Leeds LS9 7TF, UK; ICRF Cancer Medicine Research Unit, St. James's University Hospital, Leeds LS9 7TF, UK; Organon Teknika, Boxtel, The Netherlands

**Keywords:** RT–PCR, NASBA, minimal-disease, prostate, melanoma, colorectal

## Abstract

Increasingly, reverse transcriptase polymerase chain reaction (RT–PCR) is used to detect clinically significant tumour cells in blood or bone marrow. This may result in a redefinition of disease-free and clinical relapse. However, its clinical utility may be limited by lack of automation or reproducibility. Recent studies have suggested nucleic acid sequence-based amplification of target RNA may be more robust. In this study, nucleic acid sequence-based amplification was established to detect melanoma, colorectal and prostate cancer cells. Nucleic acid sequence-based amplification and RT–PCR both successfully amplified target RNA in peripheral blood samples from patients with melanoma and colorectal cancer, but only RT–PCR detected PSA in blood samples from patients with prostate cancer. There was relatively good agreement between sample replicates analyzed by RT–PCR (Kappa values of one for tyrosinase, 0.67 for CK-20 and one for PSA), but less agreement when analyzed by nucleic acid sequence-based amplification. This may limit the routine use of NASBA for the detection of clinically significant disease. In summary, RT–PCR appears at present to be the most reliable and reproducible method for the detection of low-level disease in cancer patients, although prospective studies are warranted to assess the clinical utility of different molecular diagnostic methods.

*British Journal of Cancer* (2002) **86**, 102–109. DOI: 10.1038/sj/bjc/6600014 www.bjcancer.com

© 2002 The Cancer Research Campaign

## 

The dissemination of tumour cells in patients with solid cancer is a necessary, but not sufficient, factor in the process of metastasis. Detection of dissemination is routinely used to assess the stage of disease, and subsequently define the most appropriate treatment. Consequently, sensitive and accurate methods for detection are essential to increase understanding of the role clinical metastases play in the progression of cancer, and their potential prognostic significance. Ultimately such methods will lead to a redefinition of what constitutes residual disease and clinical relapse, affecting future patient outcome.

Increasingly, reverse transcriptase polymerase chain reaction (RT–PCR) (Figure 1A

**Figure 1:**
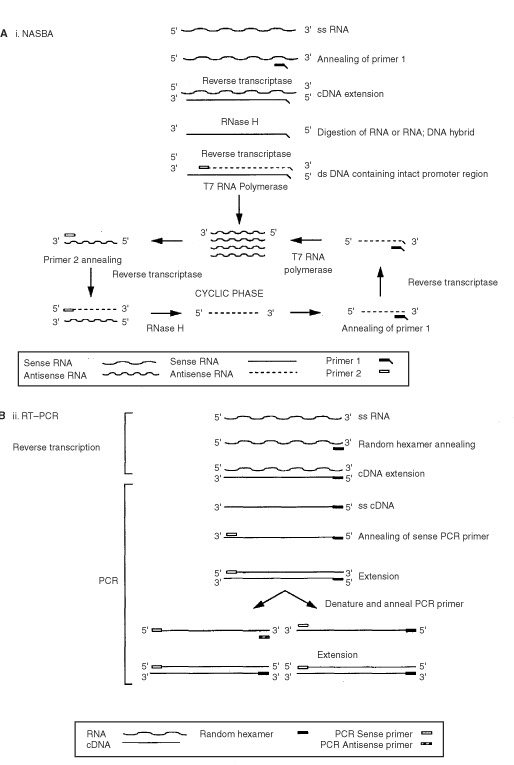
Schematic representation of methods for the amplification of mRNA using (**A**) NASBA, and (**B**) RT–PCR.

) for tumour- or tissue-specific targets is used to detect tumour cells in bone marrow, peripheral blood and stem cell harvests with greater sensitivity than more conventional methods (Johnson *et al*, 1995). There is now good evidence that using this method it is possible to detect disease of clinical significance (Burchill and Selby, 2000; Burchill *et al*, 2001). Therefore RT–PCR may increase the power of minimal residual disease and micro-metastatic disease detection in some patients with cancer, which may result in a more accurate prognosis and/or improved treatment timing or selection.

Although RT–PCR is very sensitive, its clinical utility may be limited by lack of automation and reproducibility in clinical samples, reflected in the conflicting literature and the number of reported ‘false’ positives or negatives. Previous studies have shown sample processing and the method of RNA extraction to significantly contribute to the sensitivity of RT–PCR (Keilholz *et al*, 1998; Burchill *et al*, 1999) although the question of false positive or negative results has remained a challenge. It is clear even patients with haematogenously disseminated disease do not invariably have tumour cells in all bone marrow or peripheral blood samples (Burchill and Selby, 2000). This has often been interpreted as a ‘false negative’ result, although it most likely reflects features of the metastatic disease process such as intermittent tumour-cell shedding. This emphasises the need for quality-controlled, long-term clinical outcome studies in which variables such as the frequency of sampling and sample volume are evaluated. However, false positive results remain a more technical concern. These are a particular problem when the target mRNA is contaminated with DNA, transcribed from a gene that lacks introns or where the intron-exon boundaries of the target gene are unknown.

An alternative method of RNA amplification, nucleic acid sequence-based amplification (NASBA), is reported to specifically amplify RNA but not DNA (Heim *et al*, 1998). Since NASBA amplifies RNA using an RNA T7-polymerase promoter to generate multiple RNA products at 41°C, double stranded DNA is not denatured and consequently not amplified (Figure 1B) (Chan and Fox, 1999; Simpkins *et al*, 2000). NASBA has traditionally been used for the amplification of blood-borne viruses (Kievits *et al*, 1991; van Gemen *et al*, 1993), though it may also be useful for the detection of circulating tumour cells (Lambrechts *et al*, 1998, 1999). Potentially NASBA has two powerful advantages over RT–PCR for the molecular detection of circulating tumour cells. Firstly, knowledge of the target gene intron-exon boundaries is not essential, and secondly, contaminating DNA should not affect the efficiency and reproducibility of amplification. These advantages should make the design of new amplification methods for the detection of circulating tumour cells less challenging, and improve the amplification efficiency in samples contaminated with DNA, which can be a particular problem in clinical samples.

The aims of this study were: (1) to establish NASBA assays for the detection of tumour cells; (2) compare the power of NASBA and RT–PCR to detect a mRNA target in total RNA or mixed nucleic acids (DNA/RNA); and (3) evaluate the sensitivity and specificity of circulating tumour cell detection by NASBA and RT–PCR using an *in vivo* model system and clinical samples.

## MATERIALS AND METHODS

### Cell lines

Three tumour cell lines were used to develop the NASBA assay; the colonic adenocarcinoma HT-29 (cultured in 50 : 50 DMEM : RPMI, 5% FCS), prostate carcinoma LNCAP (RPMI, 10% FCS) and malignant melanoma derived SKmel 23 (50 : 50 DMEM : RPMI, 10% FCS) (Table 1

**Table 1:**
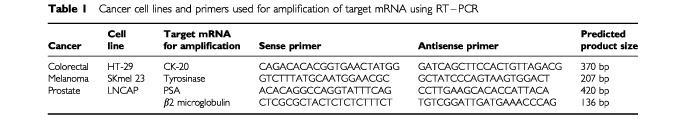
Cancer cell lines and primers used for amplification of target mRNA using RT–PCR

).

Six tumour cell lines were used as controls; the neuroblastoma cell lines SK-N-SH (50 : 50 DMEM : EMEM, 10% FCS) and IMR-32 (RPMI : DMEM, 10% FCS), the Ewing's sarcomas RD-ES and TC-32 (RPMI, 10% FCS), the rhabdomyosarcoma SJRH-30 (DMEM : RPMI, 10% FCS) and the breast carcinoma MCF-7 (DMEM, 10% FCS). Cell lines were maintained at 37°C in 5%CO_2_ : 95% air. All cell lines were purchased from the American Type Culture Collection, with the exception of the SKmel 23 cells that were a gift from Professor I Hart, Richard Dimbleby and ICRF Department of Cancer Research, St Thomas's Hospital, London.

### Extraction of total RNA

Total RNA was isolated using Ultraspec™ RNA (Biogenesis, Bournemouth, UK), as previously described (Burchill *et al*, 1994). The purity and quantity of recovered RNA was measured by reading the optical density at 260 and 280 nm. The quality of RNA was confirmed by separation of RNA (1 μg) in a 1× TBE agarose gel and staining with ethidium bromide (0.5 μg ml^−1^) before visualization under UV light, and by RT–PCR amplification for the ubiquitously expressed house-keeping gene, β2 microglobulin (see below).

### Extraction of nucleic acids (DNA/RNA)

Nucleic acids (DNA/RNA) were extracted from cell lines using the method of Boom *et al* (1990). At room temperature cells were added to 5 M guanidinium isothiocyanate, mixed and 50 μl of silica slurry added. Samples were incubated with silica for 10 min at room temperature, vortexing two or three times during this incubation. Silica was isolated by centrifugation for 15 s×13 000 **g**, and the supernatant discarded. Silica was washed twice in 950 μl of guanidinium isothiocyanate, twice in 950 μl of 70% ethanol and once in 900 μl of acetone. Silica was heated at 56°C for 10 min to dry, and subsequently the DNA/RNA was eluted in 50 μl of double distilled RNAse-free water by heating at 56°C for 10 min. The silica was isolated by centrifugation for 1 min at 13 000 **g** and the supernatant containing nucleic acids removed. This nucleic acid (DNA/RNA) mix was centrifuged a second time to remove any residual silica prior to nucleic acid spiking experiments.

### Reverse transcriptase polymerase chain reaction

Reverse transcriptase polymerase chain reaction was performed for CK-20, tyrosinase, PSA and β2 microglobulin using primers designed to amplify across an intron/exon boundary (Table 1) as previously described (Burchill *et al*, 1994). Briefly, RNA (in 5 μl of depc treated water) was heated to 95°C for 5 min and rapidly cooled on ice. Reaction mix (5 μl) was added to the RNA to give final concentrations of 1× PCR buffer (50 mM KCl, 10 mM Tris HCl, pH 8.3), 1 mM dATP, dCTP, dGTP and dTTP (Pharmacia, Uppsala, Sweden), 8 mM MgCl_2_, 15 ng random hexamer primers (Gibco BRL), 20 U RNA guard (Pharmacia) and 5 U murine moloney leukaemia virus reverse transcriptase (Pharmacia). After incubation at 37°C for 1 h the samples were heated to 95°C for 5 min to destroy the reverse transcriptase (RT) activity. After cooling on ice, 40 μl of PCR mix (1× PCR buffer as above, 2.5 U Amplitaq Gold (Perkin Elmer) and 25–50 mM of each primer (Table 1)) were added to the RT products, mixed and overlaid with mineral oil.

Tyrosinase was amplified by denaturation at 95°C for 1 min, annealing at 55°C for 1 min and extension at 72°C for 1 min for 50 cycles, CK-20 and PSA mRNA were amplified under the same conditions except the annealing temperature was increased to 60°C. A final extension for 10 min at 72°C was performed for each target. To ensure that only RNA was amplified, a negative control in which the RT enzyme was omitted was performed for each sample. After amplification, the PCR products were separated by electrophoresis in a 2% agarose gel, stained with ethidium bromide (0.5 μg ml^−1^) and visualized under UV light. The identity of amplified products was initially confirmed by direct sequence analysis, and by Southern blotting in subsequent experiments. Primers for RT–PCR were purchased from the Imperial Cancer Research Fund, Lincoln's Inn Fields, London.

### Nucleic acid sequence-based amplification

Nucleic acid sequence-based amplification assays were established using cell line RNA at the laboratories of Organon Teknika (Boxtel, The Netherlands). Briefly, multiple primer and probe sets for each of the mRNA targets were developed and tested in serial dilutions of cell line RNA. The optimal KCL concentration and amplicon dilution factors were determined individually for each target. In these optimization studies amplified products were Northern blotted and hybridized with ^32^P-labelled probes; the best primer/probe combination was used to develop the NASBA ECL detection assay (Table 2

**Table 2:**
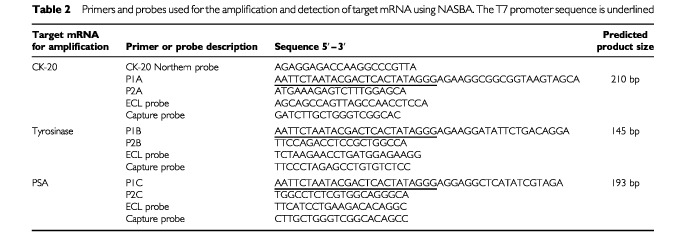
Primers and probes used for the amplification and detection of target mRNA using NASBA. The T7 promoter sequence is underlined

). All subsequent experiments were carried out in Leeds using these optimized conditions.

Briefly, RNA (in 5 μl) was added to 10 μl of NASBA buffer and pre-incubated at 65°C for 5 min before incubation at 41°C for 5 min. Enzyme mix (5 μl; 0.08 U *E.coli* RNase H, 32 U T7-RNA polymerase, 6.4 U avian myeloblastosis virus RT) was added and the reaction was incubated at 41°C for 90 min. The final concentrations in the NASBA buffer were 40 mM Tris HCl pH 8.5, 12 mM MgCl_2_, 70 mM KCl, 5 mM dithiothreitol, 15% dimethyl sulphoxide, 1 mM of each dNTP, 2 mM of ATP, CTP and UTP, 1.5 mM GTP, 0.5 mM ITP and 10 pM of each primer. Primer sets used for amplification are shown in Table 2.

A semi-automated electro-chemiluminescense system (NASBA QR) was used to detect amplicons. Products were diluted and hybridized with two probes complementary to the amplified RNA sequence in pre-mix (Table 2). The biotinylated capture probe is immobilized onto streptavidin coated paramagnetic beads, and the ECL probe is labelled with ruthenium ester, the fluorescence of which is detected by the NucliSens® ECL reader (Organon Teknika). The pre-mix contained 10 μl of 0.05 mg beads and 0.084 μM of biotinylated capture probe in 1× PBS and 0.1% BSA, and 10 μl of 0.05 μM ruthenium probe in 5 g l^−1^ 2-chloro acetamide, 0.1% BSA and 12.5× SSC. The optimum dilution of amplified products and temperature of annealing are different for each target. For CK-20 and tyrosinase the optimum dilution factor is 1 : 16, for PSA 1 : 2; annealing was performed at 50°C, 41°C and 60°C respectively.

A proprietary reference positive control was included in each assay, along with a hybridization assay negative control (consisting of the two probes with water instead of the amplification reaction components). The hybridization negative control was used to define a cut-off for ECL readings; any reading 10 times greater than the negative control value was taken as positive. The probes and primers for NASBA were synthesized and purified by Organon Teknika.

### *In vitro* sensitivity, specificity and reproducibility

The sensitivity of NASBA and RT–PCR for the detection of target mRNA in a background of total RNA or total nucleic acids was evaluated by diluting RNA or nucleic acids (DNA/RNA) from HT-29 cells (which express CK-20 mRNA), in either total RNA or nucleic acids (DNA/RNA) from a cell line that does not express CK-20 mRNA (SK-N-SH). RNA (0–1 ng, extracted using Ultraspec™) or DNA/RNA (0–1 ng, isolated using the Boom method) from HT-29 cells was added to RNA (5 μg) or DNA/RNA (5 μg) extracted from the neuroblastoma cell line SK-N-SH. These spiked samples were prepared and analyzed for CK-20 mRNA by NASBA and RT–PCR three times. Data was analyzed using exact logistic regression (Mehta and Patel, 1995).

The sensitivity, specificity and reproducibility of NASBA and RT–PCR for the detection of CK-20, PSA and tyrosinase mRNA were subsequently evaluated using RNA spiking experiments. RNA (0–100 ng) extracted from HT-29, LNCAP and SKmel 23 cells using Ultraspec™, was added to 5 μg of RNA extracted from normal peripheral blood of volunteers. Parallel samples were analyzed for CK-20, PSA or tyrosinase mRNA in six separate experiments. The reproducibility of, and agreement between NASBA and RT–PCR were assessed by calculating the Kappa statistic (Altman, 1991). The Kappa statistic represents the level of agreement observed over and above that expected by chance. It is defined as (Probability observed−Probability expected) ÷ (1 – Probability expected). It can vary between −1 and +1, with 0 representing no agreement over that expected by chance. A high level of agreement is usually indicated by a Kappa of approximately 0.7 or above.

### Clinical samples

Blood samples were collected from patients with melanoma (*n*=12), colon (*n*=12) or prostate cancer (*n*=12) attending the ICRF Medical Oncology Unit, St James's University Hospital, Leeds or Cookridge Hospital, Leeds. Normal control blood samples (*n*=8) were taken from healthy volunteers. All blood samples (2 ml) were collected into 20 mM EDTA, mixed and 8 ml of Ultraspec™ (Biogenesis, Bournemouth, UK) added. Samples were stored at −80°C until required for RNA extraction. Each RNA sample was divided and equivalent to 1 μg of RNA analyzed by NASBA or RT–PCR. All patients and volunteers from whom blood was taken gave informed consent.

## RESULTS

### NASBA assay for CK-20, PSA and tyrosinase

**Figure 2:**
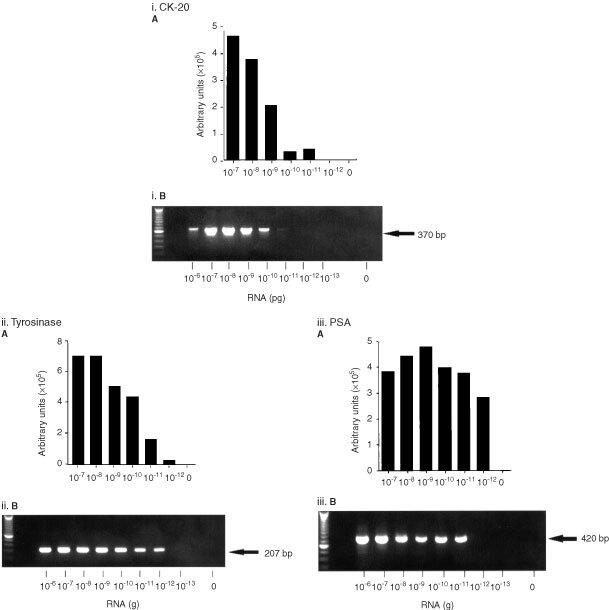
Amplification of (i) CK-20; (ii) tyrosinase; and (iii) PSA mRNA in total RNA extracted from HT-29, SKmel 23 and LNCAP cell lines respectively using NASBA (**A**) and RT–PCR (**B**). A histogram showing the ECL read-outs for NASBA, and RT–PCR products separated in an agarose gel, stained with ethidium bromide and visualized under UV light are shown. The identity of RT–PCR amplified products was confirmed by both Southern blot and direct sequence analysis (results not shown).

A NASBA assay was established for the specific detection of CK-20, PSA and tyrosinase mRNA in RNA extracted from HT-29, LNCAP and SKmel-23 cells respectively (Figure 2

). Target mRNA was not detected in RNA from control cell lines, or in RNA extracted from peripheral blood of healthy volunteers. Using NASBA it was possible to detect CK-20 mRNA in 10 pg of total RNA from HT-29 cells, and PSA and tyrosinase mRNA in 1 pg of LNCAP and SKmel 23 RNA respectively (Figure 2).

### Sensitivity of NASBA and RT*–*PCR for the detection of CK-20 mRNA in isolated RNA and nucleic acids (DNA/RNA)

The detection of CK-20 mRNA (1000 pg of HT-29 RNA) diluted in unrelated RNA or nucleic acids (DNA/RNA) by RT–PCR or NASBA was equally sensitive (24 out of 24 positive) (Table 3

**Table 3:**
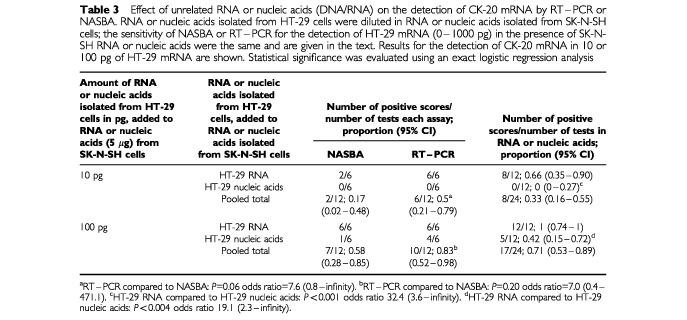
Effect of unrelated RNA or nucleic acids (DNA/RNA) on the detection of CK-20 mRNA by RT–PCR or NASBA. RNA or nucleic acids isolated from HT-29 cells were diluted in RNA or nucleic acids isolated from SK-N-SH cells; the sensitivity of NASBA or RT–PCR for the detection of HT-29 mRNA (0–1000 pg) in the presence of SK-N-SH RNA or nucleic acids were the same and are given in the text. Results for the detection of CK-20 mRNA in 10 or 100 pg of HT-29 mRNA are shown. Statistical significance was evaluated using an exact logistic regression analysis

). Furthermore both NASBA and RT–PCR were equally specific, 0 out of 24 tests were positive for CK-20 mRNA when no target mRNA was added. The addition of 5 μg of RNA or nucleic acids isolated from SK-N-SH cells, which do not express CK-20 mRNA, did not affect the specificity of amplification (*P*>0.5, results not shown). Therefore the amplification results for CK-20 mRNA detection by NASBA or RT–PCR in the presence of SK-N-SH derived RNA or nucleic acids have been pooled (Table 3). However, the number of samples positive for CK-20 mRNA (8 out of 12) detected in HT-29 RNA diluted in total RNA was significantly higher than the number of positives detected in nucleic acids (DNA/RNA) (for 10 pg of HT-29 RNA *P*<0.001 odds ratio 32.4, 95% CI 3.6–infinity and for 100 pg of HT-29 RNA *P*=0.004 odds ratio 7.0, 95% CI 0.4–471.1 respectively) (Table 3).

At a dilution of 10 pg of HT-29 RNA, RT–PCR detected CK-20 mRNA in 6 out of 12 samples compared to 2 out of 12 detected by NASBA (*P*=0.06), odds ratio 7.6 (95% CI 0.8–infinity); whereas at 100 pg of HT-29 RNA RT–PCR detected 10 out of 12 positive samples compared to 7 out of 12 for NASBA (*P*=0.20, odds ratio 7.0 (95% CI 0.4–471.1) (Table 3).

### Sensitivity and reproducibility of NASBA and RT*–*PCR assays for detection of CK-20, PSA and tyrosinase mRNA

#### *In vitro* cell assay

The sensitivity of NASBA for the detection of tumour cell derived target mRNA is similar to that achieved with RT–PCR, the absolute sensitivity of either assay varying depending on the target mRNA species. The maximum sensitivity for the detection of CK-20 mRNA by NASBA was comparable to that for its detection by RT–PCR (Table 4

**Table 4:**
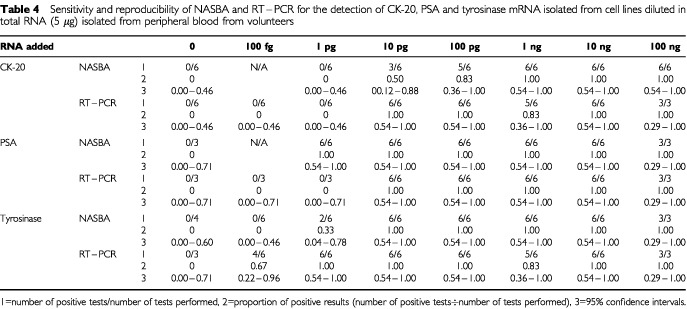
Sensitivity and reproducibility of NASBA and RT–PCR for the detection of CK-20, PSA and tyrosinase mRNA isolated from cell lines diluted in total RNA (5 μg) isolated from peripheral blood from volunteers

). However the NASBA assay for the detection of PSA was an order of magnitude more sensitive than RT–PCR, whereas the NASBA assay for tyrosinase was an order of magnitude less sensitive than RT–PCR (Table 4). The maximum sensitivity range for NASBA or RT–PCR for all three mRNA targets in the cell lines used was between 100 fg and 10 ng.

#### Patient blood samples

NASBA and RT–PCR both successfully amplified target mRNA in peripheral blood from patients with melanoma and colorectal cancer (Table 5

**Table 5:**
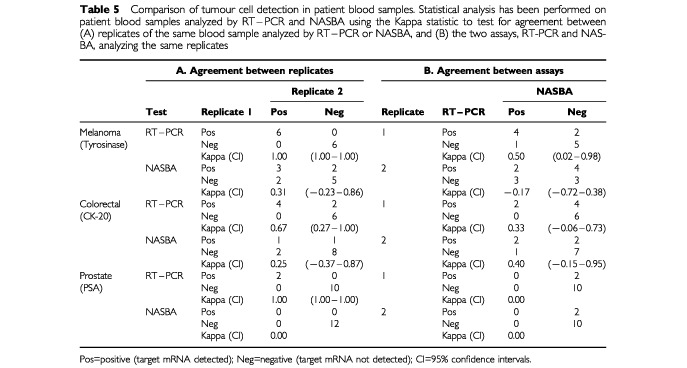
Comparison of tumour cell detection in patient blood samples. Statistical analysis has been performed on patient blood samples analyzed by RT–PCR and NASBA using the Kappa statistic to test for agreement between (A) replicates of the same blood sample analyzed by RT–PCR or NASBA, and (B) the two assays, RT-PCR and NASBA, analyzing the same replicates

). However NASBA failed to detect PSA mRNA, detected by RT–PCR, in peripheral blood from two patients with prostate cancer (Table 5).

There was good agreement between sample replicates analyzed by RT–PCR for tyrosinase, CK-20 or PSA mRNA, as demonstrated by the Kappa values of 1, 0.67 and 1 respectively (Table 5). However replicates analyzed by NASBA for tyrosinase or CK-20 mRNA showed no statistical agreement (Table 5). PSA mRNA was not detected by NASBA in any of the blood samples from patients with prostate cancer (Table 5). Since both observed agreement proportions and expected agreement proportions were equal to 1, the Kappa (agreement above that expected by chance) is either 0 or undefined; providing little information on the expected level of agreement.

There was no statistical agreement, beyond chance, between the presence of CK-20, tyrosinase or PSA mRNA in peripheral blood when analyzed by NASBA and RT–PCR. This is not so surprising given the lack of agreement between replicates analyzed by NASBA (Table 5).

## DISCUSSION

NASBA can be used to detect mRNA transcripts in tumour cell lines to a sensitivity equal to that detected by RT–PCR. The sensitivity of both methods is target dependent, and requires careful primer design and assay optimization. All the assays used in this study were specific, and did not amplify unrelated RNA's in either control blood samples or control tumour cell lines. However, during the development of the NASBA assay for CK-20 and tyrosinase mRNA primer sets used within a single exon amplified target products in whole blood and human placental nucleic acids (results not shown). The development of both NASBA and RT–PCR assays required knowledge of the gene sequences and their intron-exon boundaries, allowing design of primers that amplify across introns to prevent generation of products from contaminating DNA. Previous studies have suggested the nucleic acid isolation procedure has no influence on the amplification by NASBA (van Gemen *et al*, 1995; Chan and Fox, 1999; Simpkins *et al*, 2000), however when used to detect low copy number this clearly becomes a significant factor.

The clinical application of RT–PCR or NASBA for the detection of circulating tumour cells requires a reliable and reproducible assay. Although at higher concentrations of target mRNA (>10 ng) both assays are reliable and reproducible when analyzing target cell RNA spiked samples, at lower dilutions (1 : 5×10^7^ or 1 : 5×10^6^) the assays become less robust. This suggests detection of tumour derived RNA is at the limits of the assay sensitivity, consequently it is not surprising that the reproducibility between replicates is not consistent at these low levels. However analysis of blood samples from patients with melanoma or colorectal cancer by NASBA was less informative than analysis by RT–PCR. Furthermore, NASBA failed to detect tumour cells in blood samples from patients with advanced prostate cancer, although by RT–PCR two of the blood samples analyzed were positive. The lack of concordance between duplicates when analyzed by NASBA indicates the difficulties in assessing this assay at the limits of detection. It would appear from the current study RT–PCR is more robust than NASBA for the detection of low-level target RNAs.

The sensitivity of both NASBA and RT–PCR was increased when CK-20 mRNA was detected in isolated RNA rather than isolated nucleic acids (DNA/RNA). This is not surprising as the amount of CK-20 mRNA in 10 pg of purified RNA will be greater than in 10 pg of nucleic acid (DNA/RNA). Previous studies have shown the method of processing samples affects the sensitivity of target gene detection by RT–PCR (Burchill and Selby, 2000), it would appear this may also be the case with NASBA. Interestingly the sensitive detection of viral RNA by NASBA is usually carried out on total nucleic acids (van Gemen *et al*, 1995; Damen *et al*, 1999; Fiscus *et al*, 2000). Whether detection of viral RNA would be more sensitive if RNA was purified is not clear. When target RNA is abundant the method of sample processing is less critical, however at the limits of detection purification of RNA prior to assay may be beneficial.

Analysis of amplicons using NucliSens®, like realtime RT–PCR (Lie and Petropoulos, 1998; Bremer *et al*, 2000), has the advantage over more traditional read-outs that sequence specific probes are used to detect amplified products. Consequently the identity of amplified products is confirmed without the need for additional steps (such as Northern blot). The products of NASBA can also be analyzed in a quantitative way using a NucliSens® reader and internal standards (van Gemen *et al*, 1995; Bustin, 2000), however the NucliSens® reader detects amplified products at an end-point unlike real-time PCR that detects products as they are generated. Although both methods can be used to quantify the number of RNA transcripts per sample, neither accurately reflects cell number since the number of target RNA molecules per tumour cell is not known. With increased knowledge of RNA transcripts per tumour cell it may be possible to use such methods to assess cell number. This is likely to be more informative within an individual than between patients, as the level of RNA transcripts per cell varies. However, changes in gene transcription (induced by such factors as chemotherapy) may limit this (Burchill, unpublished observations).

In summary, NASBA can be used to detect low-levels of tumour-derived target RNA. However despite the single tube approach and semi-automation, lack of reproducibility currently limits its potential application for the detection of clinically relevant disease in patients with cancer. Amplification of tumour RNA in peripheral blood by RT–PCR currently appears to be the most reproducible method for the detection of low-level disease in cancer patients. However this method is labour intensive, and would benefit from the development of an automated procedure that could be used in clinical outcome studies.
